# Cholesterol-Rich Lipid Rafts as Platforms for SARS-CoV-2 Entry

**DOI:** 10.3389/fimmu.2021.796855

**Published:** 2021-12-16

**Authors:** Selvin Noé Palacios-Rápalo, Luis Adrián De Jesús-González, Carlos Daniel Cordero-Rivera, Carlos Noe Farfan-Morales, Juan Fidel Osuna-Ramos, Gustavo Martínez-Mier, Judith Quistián-Galván, Armando Muñoz-Pérez, Víctor Bernal-Dolores, Rosa María del Ángel, José Manuel Reyes-Ruiz

**Affiliations:** ^1^ Department of Infectomics and Molecular Pathogenesis, Center for Research and Advanced Studies (CINVESTAV-IPN), Mexico City, Mexico; ^2^ Unidad Médica de Alta Especialidad, Hospital de Especialidades No. 14, Centro Médico Nacional “Adolfo Ruiz Cortines”, Instituto Mexicano del Seguro Social (IMSS) Veracruz Norte, Veracruz, Mexico

**Keywords:** COVID-19, SARS-CoV-2 attachment and entry, cholesterol-rich lipid rafts, immune response, antiviral therapy

## Abstract

Since its appearance, the Severe Acute Respiratory Syndrome Coronavirus (SARS-CoV-2), the causal agent of Coronavirus Disease 2019 (COVID-19), represents a global problem for human health that involves the host lipid homeostasis. Regarding, lipid rafts are functional membrane microdomains with highly and tightly packed lipid molecules. These regions enriched in sphingolipids and cholesterol recruit and concentrate several receptors and molecules involved in pathogen recognition and cellular signaling. Cholesterol-rich lipid rafts have multiple functions for viral replication; however, their role in SARS-CoV-2 infection remains unclear. In this review, we discussed the novel evidence on the cholesterol-rich lipid rafts as a platform for SARS-CoV-2 entry, where receptors such as the angiotensin-converting enzyme-2 (ACE-2), heparan sulfate proteoglycans (HSPGs), human Toll-like receptors (TLRs), transmembrane serine proteases (TMPRSS), CD-147 and HDL-scavenger receptor B type 1 (SR-B1) are recruited for their interaction with the viral spike protein. FDA-approved drugs such as statins, metformin, hydroxychloroquine, and cyclodextrins (methyl-β-cyclodextrin) can disrupt cholesterol-rich lipid rafts to regulate key molecules in the immune signaling pathways triggered by SARS-CoV-2 infection. Taken together, better knowledge on cholesterol-rich lipid rafts in the SARS-CoV-2-host interactions will provide valuable insights into pathogenesis and the identification of novel therapeutic targets.

## Introduction

The current Coronavirus disease 2019 (COVID-19) emergency is considered a global health threat (1). COVID-19 includes dyspnea, fever, headache, myalgia, and severe outcomes such as severe pneumonia, respiratory failure, multiple organ failure, including death (2). Severe Acute Respiratory Syndrome Coronavirus (SARS-CoV-2), the causal agent of COVID-19, belongs to the family of *Coronaviridae* (3) and is translated into four structural proteins (S, E, M, and N) and sixteen non-structural proteins (NSP1−16) (4). The structural S protein or spike glycoprotein-mediated the coronavirus entry into host cells and comprised two subunits (5). The S1 subunit poses a receptor-binding domain (RBD) that specifically interacts with the host cell ACE-2 receptor, and the S2 subunit fuses the membranes of viruses and host cells (5). ACE-2 is a homolog of ACE-1 receptor that mediates the angiotensin II production to activate the renin-angiotensin system (RAS) and plays a crucial role in cardiovascular diseases (6). The ACE-2 receptor is widely expressed in the heart, lung, and kidney (7) and functions during SARS-CoV-2 entry (8). Moreover, new evidence demonstrates the involvement of other receptors and cholesterol-rich lipid rafts in the SARS-CoV-2 internalization (9–17).

Due to the lipid rafts containing proteins and high concentrations of sphingolipids and cholesterol, the plasma membrane is less fluid than the rest (18). Thus, these rigid domains in the cell membrane provide a platform for diverse receptors involved in cell signaling and other functions (19–22). In addition, the lipid rafts contain specific receptors that mediate the internalization of pathogens through distinct entry mechanisms and modulate the lipid raft-dependent immune response (23, 24).

During SARS-CoV-2 infection, receptors relying on cholesterol-rich lipid rafts that contribute to the progression of inflammation are involved in viral entry (9, 11, 15, 16). Syndecans, a protein of the transmembrane proteoglycan family, facilitate the SARS-CoV-2 entry (15). Moreover, the SARS-CoV-2 S protein interacts with heparan sulfate and ACE-2 at the cell surface (10). Interestingly, receptors involved in the immune system, such as CD-147 and human Toll-like receptors (TLR), play an essential role in host cell entry and activation of the innate immune response to SARS-CoV-2 (11, 25). Hence, cholesterol-rich lipid rafts play an essential role in regulating the immune response and targeting antiviral therapy during SARS-CoV-2 infection.

The role of cholesterol-rich lipid rafts in the viral entry, assembly, and release was elucidated using cholesterol-lowering treatments (26–28). The efficient removal of cholesterol from these membrane microdomains leads to a disruption of the signaling pathways regulated by lipid rafts and the elimination of proteins associated with them (27, 29–31). In SARS-CoV-2 entry, the integrity of cholesterol-rich lipid rafts can modulate the interaction between the receptor and viral S protein (32). However, a better understanding of the role of cholesterol-rich lipid rafts in the host-SARS-CoV-2 interaction will provide valuable insights into novel mechanisms of viral entry and the development of new and alternate antiviral therapies.

## Lipid Rafts and Coronaviruses

Membrane microdomains enriched in cholesterol and glycosphingolipids are called lipid rafts (18). The cholesterol-rich lipid rafts concentrate cellular proteins and lipids (19–22). In addition, cholesterol-rich lipid rafts are crucial cellular factors involved in viral replication (31, 33). The concentration of receptors and co-receptors in cholesterol-rich lipid rafts facilitates the virus fusion with host cell membranes, promoting efficient viral entry (31, 33, 34).

Coronaviruses are diverse viruses that infect a broad range of organisms (35). Human coronaviruses (HCoV-229E, HCoV-OC43, HCoV-NL63, and HCoV-HKU1) circulate worldwide, causing seasonal and usually mild respiratory tract infections (36). However, SARS-CoV, Middle East respiratory syndrome coronavirus (MERS-CoV), and SARS-CoV-2 are highly pathogenic, producing life-threatening respiratory pathologies and lung injuries (35, 37).

The cholesterol in the lipid rafts is indispensable for the internalization of the coronaviruses (38–41). Interestingly, cholesterol depletion from lipid rafts with cholesterol-lowering treatments such as methyl-β-cyclodextrin (MβCD) affects the interaction between the SARS-CoV S protein and the ACE-2 receptor (42). Also, a decrease of ACE-2 on the cell surface and reduction of the SARS-CoV entry was determined in MβCD pretreated cells (43). Therefore, based on their research, Glende et al. suggested that the difference in these data is due to their protocols, speculating that cholesterol-rich lipid rafts affect ACE-2 protein conformation and the presentation of antigenic epitopes (42). Furthermore, the depletion of this cholesterol affects the spatial localization of ACE-2 in lipid rafts, demonstrating the importance of cholesterol-rich lipid rafts for efficient interaction between the viral surface protein and the cellular receptor (42).

Although Coronavirus envelope (E) protein is not related to viral entry, this protein plays a prominent role in viral morphogenesis and pathogenesis (44). SARS-CoV E protein can be translocated from the Golgi apparatus and endoplasmic reticulum to the cell surface, specifically in cholesterol-rich lipid rafts (45, 46). The E protein transmembrane domain is associated with membrane permeabilizing activity and inflammasome activation (45, 46). Since E protein is highly conserved among coronaviruses, this could be related to the membrane ion channel activity in the SARS-CoV-2 pathogenesis (47). The cholesterol depletion from lipid rafts decreases the virus entry and contributes to altering the membrane protein composition (43, 48); therefore, this process could impact the pathogenic mechanisms involving the E protein.

Overall, these results indicate that cholesterol-rich lipid rafts are required for entry and pathogenesis of the coronaviruses.

## The Role of Cholesterol-Rich Lipid Rafts in SARS-CoV-2 Entry

**Figure 1:**
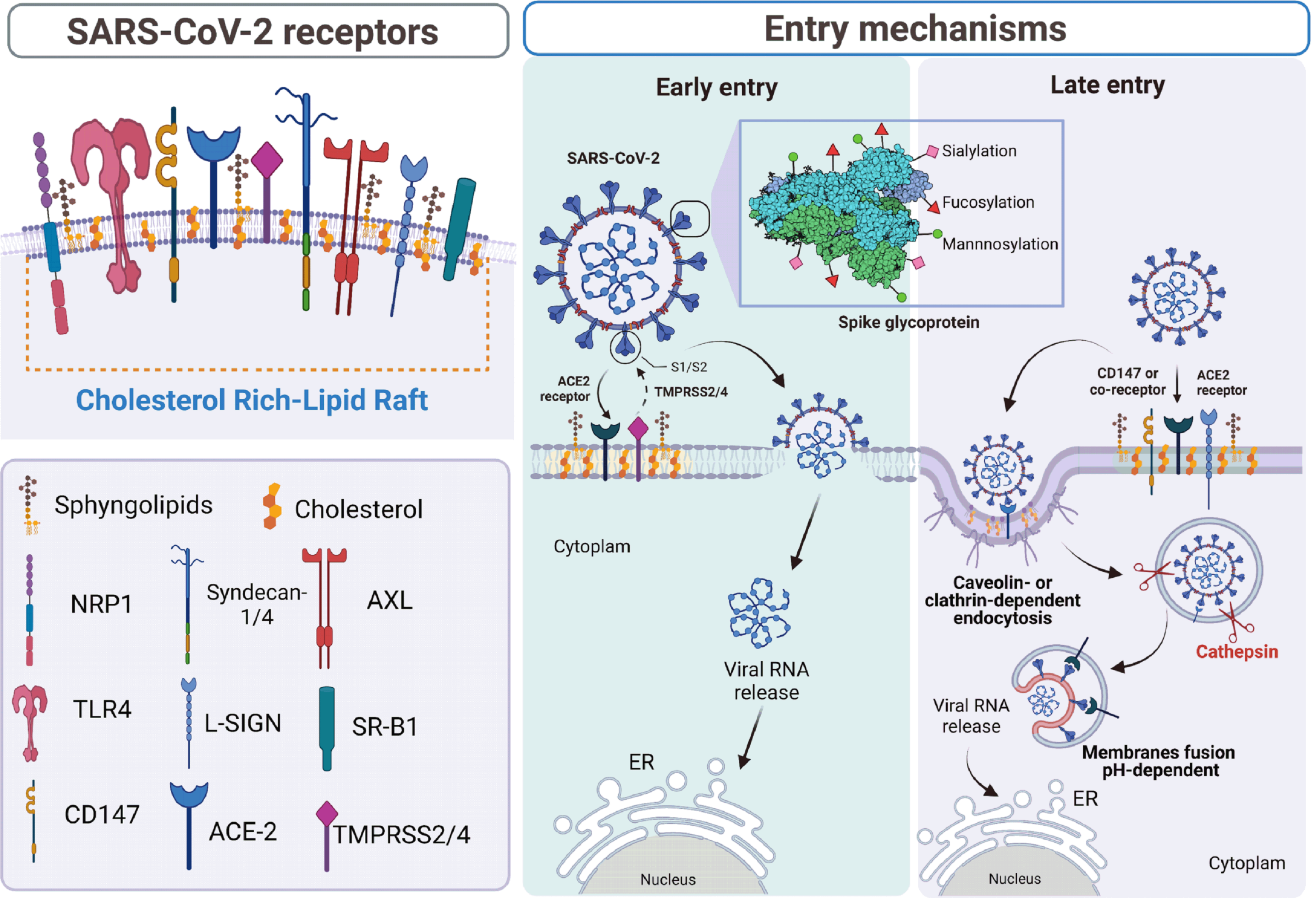
Schematic representation of SARS-CoV-2 viral entry *via* receptors located on cholesterol-rich lipid rafts. In the left panel, we represent the different receptors and co-receptors located in the cholesterol-rich lipid rafts, described to participate and enhance the entry of SARS-CoV-2 (50). In the right panel, we represent the post-translational modifications of the spike protein enveloped in the attachment and the early and late entry of the virus. Early entry involves the major receptor ACE-2 and the transmembrane protease TMPRSS2/4 that promotes pH-independent activation of the spike protein, which exposes the fusion peptide allowing fusion between cell and viral membranes (13, 51, 52). Late entry involves the ACE-2 receptor and a co-receptor such as HSPG (10), Syndecan-1/4 (15), NRP1 (53), L-SIGN (16), and SR-B1 (12). On the other hand, during the absence of ACE-2, the receptors CD-147 (11), AXL (14, 17), and probably TLR4 (9) activate the endocytic pathway mediated by clathrin or caveolin. In this endosomal compartment, the SARS-CoV-2 S protein is activated by the pH-dependent protease cathepsin, releasing the RNA into the cell cytoplasm (8). The graphical was elaborated using BioRender.com.

SARS-CoV-2 is a positive-sense RNA virus with two large overlapping open reading frames (ORF1a and ORF1b). This large single-stranded RNA genome of ~ 30,000 nt encodes two large polyproteins, pp1a (440-500 kDa) and pp1ab (740-810 kDa), which are cleaved into the NSP1 to 11 and NSP12 to 16, respectively (4). Some NSP contain functional domains, including the 3C-like cysteine proteinase (3CL^pro^, NSP5), RNA-dependent RNA polymerase (RdRp, most of NSP12), nidovirus RdRp-associated nucleotidyltransferase (N terminal of NSP12), helicase (Hel, NSP13), and exonuclease (ExoN, NSP14) (4, 49). Also, the SARS-CoV-2 genome encodes four structural proteins: spike surface glycoprotein (S), envelope (E), membrane (M), and nucleocapsid (N) that are essential for viral entry and assembly; and nine accessory proteins involved in the host immune response during infection (4, 49). SARS-CoV-2 attachment is the first step in the infection process, where the S protein on the envelope of the virus recognizes the host cell receptors and mediates the viral entry (**Figure 1**) (49). S protein is cleaved by cellular proteases into the S1 and S2 subunits to give rise to trimers of the S1/S2 heterodimer (**Figure 1**) (54, 55). The S1 subunit contains the N-terminal domain (NTD) and the C-terminal domain (CTD), also called the receptor-binding domain (RBD), since it domain is responsible for binding the host receptor ACE-2 (54–56). The tip of the NTD of the SARS-CoV-2 S protein has a ganglioside-binding domain (52 amino acid residues, 111-162) that could enhance virus attachment to lipid rafts in the host cell membrane and facilitate receptors binding (57), which will be discussed below. Moreover, the cleavage of SARS-CoV-2 S protein at the S1-S2 site produces the sequence TQTNSPRRAR_OH_, the binding site for neuropilin 1 (NRP1), an entry receptor found in the olfactory neuronal cells (53).

After attachment, the entry to the host cells of SARS-CoV-2 occurs *via* clathrin-mediated endocytosis in multiple cell types such as VERO, A549, and HEK-293T cells (58). In contrast, the caveolin-, clathrin-, endophilin A2-mediated endocytosis, and macropinocytosis might not be involved in SARS-CoV-2 entry (59). Bayati et al. used a Lentivirus pseudotyped with purified SARS-CoV-2 S protein prefusion-stabilized ectodomain (58). Therefore, the SARS-CoV-2 entry into the cell could depend on the distinct conformational states of the S protein (**Figure 1**) (60, 61).

The clathrin-independent carriers/GPI-anchored-protein-enriched early endosomal compartments (CLIC/GEEC) is an essential pathway for cholesterol-rich lipid raft components (62, 63). Li et al. demonstrated that the CDC42-involved CLIC/GEEC pathway is unlikely to participate in the infection of SARS-CoV-2 (59). Nevertheless, SARS-CoV-2 entry to host cells is cholesterol-rich lipid rafts dependent (59). Two pathways can regulate it: (1) the membrane fusion using proteases such as TMPRSS2, and (2) the endosomal pathway (where the cathepsin B and L (CatB/L) is involved), which is less efficient than the fusion pathway (**Figure 1**) (64, 65). In this sense, CatL but not CatB facilities SARS-CoV-2 entry (**Figure 1**). When the TMPRSS2 expression is absent, the CatL is critical to viral entry mediated by S protein; however, if TMPRSS2 is expressed, the use of CatL is markedly diminished (66). The proteolytic activation by CatL depends on endosomal acidification and is inhibited when the endosomal pH increases (**Figure 1**) (66). In contrast, the TMPRSS2-mediated entry pathway is not affected by pH, and it is more dependent and preferred over the CatL-mediated pathway for SARS-CoV-2 (66). Ou et al. also demonstrated that the furin-cleavage in the SARS-CoV-2-producing cell correlates with greater dependence on TMPRSS2 and lower dependence on CatL (66).

On the other hand, the cells from Nieman-Pick disease type C (NPC), a lysosomal storage disorder, have reduced lipid rafts. This could create unfavorable environments for SARS-CoV-2 infectivity (67). Hence, Ballout et al. hypothesized that the NPC cells might affect the trafficking of SARS-CoV-2 receptors such as ACE-2 and block SARS-CoV-2 fusion by the CatL leakage or affecting the proteolytic activity of CatL when the intra-lysosomal pH increases (67). In this regard, Nieman-Pick disease type C1 (NPC1) receptor, an endosomal membrane protein that regulates intracellular cholesterol traffic, interacts with SARS-CoV-2 N protein (68). The mechanism by which the SARS-CoV-2 N protein interacts with NPC1 is unknown. One possibility is that similarly to the interaction between the Ebola virus (EBOV)-glycoprotein (GP) and NPC1, SARS-CoV-2 could traffic the endocytic pathway for viral uncoating through the fusion of late endosomes and lysosomes (68, 69). It is not yet clear the specific role of NPC1 during SARS-CoV-2 infection. However, the antiviral compounds that interact with NPC1, such as carbazole SC816 and sulfides SC073 and SC198 (drugs used to elucidate the interaction between EBOV-GP and NPC1) can reduce SARS-CoV-2 infection with a good selectivity index in human cell infection models (68, 70). This receptor was found in a genome-scale CRISPR loss-of-function screen performed to identify host factors required for SARS-CoV-2 viral infection of human alveolar basal epithelial carcinoma cells (71). Thus, the role of NPC1 in cholesterol regulation is essential during SARS-CoV-2 infection (67, 71, 72). Also, the panel of the top-ranked genes screened by Daniloski et al. revealed that RAB7A regulates cell surface expression of ACE-2, likely by sequestering this SARS-CoV-2 receptor in endosomal vesicles (71). RAB7A interacts with the SARS-CoV-2 NSP7 protein (73), a viral protein required for the RdRP complex assembly (74). Since the RAB7A is required for exosome secretion (75), SARS-CoV-2 could use an exosome pathway as a route of entry or egress, similar to other viruses (76). Exosomes isolated from COVID-19 patients contain the SARS-CoV-2 RNA and proteins implicated in the exosomal cargo (77). These results suggest that exosomes are involved in the mechanisms associated with tissue damage and multiple organ injury in COVID-19 patients (77).

Upon cell entry, cholesterol-rich lipid rafts found on the outer leaflet of the plasma membrane can play an essential role in membrane fusion between the SARS-CoV-2 particle and the early endosome to allow the viral genome to be released into the cytoplasm (**Figure 1**). In this sense, the upstream helix (UH) region is removed by S2-proteolytic cleavage to activate irreversible conformational changes and initiate membrane fusion (49, 60). This SARS-CoV-2 replication step depends on the cholesterol-rich lipid rafts and endosomal acidification. The cholesterol-rich lipid rafts are found on the luminal side of the endosome (78–80). Glycerophospholipid bis(monoacylglycerol)phosphate (BMP) is also named lysobisphosphatidic acid (LBPA) (81). BMP is enriched in the internal membranes of the late endosome/lysosome, regulating cholesterol distribution on the lipid rafts (81). The accumulation of BMP reduces the expression of ATP-binding cassette transporter G1 (ABCG1), a lipid transporter responsible for lung lipid homeostasis and acting as a protective factor during infections (82, 83). Cholesterol-rich lipid rafts are determinants for SARS-CoV-2 interaction with the cellular receptor ACE2 (**Figure 1**) (59). BMP regulation of these cholesterol-rich membrane domains could impact viral entry (81). BMP regulates the cholesterol efflux to HDL in macrophages and the production of oxysterols such as 25-Hydroxycholesterol (25-HC) (84). 25-HC is a potent inhibitor of SARS-CoV-2 replication by restricting the S protein catalyzed membrane fusion *via* blockade of cholesterol export in the late endosomes (85). 25-HC is the product of cholesterol oxidation by the enzyme cholesterol-25-hydroxylase and can control sterol biosynthesis by regulating Sterol Regulatory Element Binding Protein (SREBP) (86). Zu et al. suggested that 25-HC could be considered a risk marker for the severity due to its high concentration in a fatal COVID-19 patient and SARS-CoV-2 infected hACE2 mice (87). 27-hydroxicholesterol (27-HC) is accumulated in plasma membrane lipid rafts when exogenously added (88). 27-HC could have important implications for modifying the structure and function of membrane lipid-protein clusters during SARS-CoV-2 infection (88). Although the SARS-CoV-2 particle is not directly inactive by 27-HC, this modifies cell structures of the lipid rafts by the accumulation of cholesterol, inducing a transient modification of the endosomal membrane composition and function to inhibit SARS-CoV-2 replication (88). Interestingly, Marcello et al. demonstrated that blood levels of 27-HC were decreased in patients with severe COVID-19 (88). Also, they showed an increased serum level of 7-ketocholesterol and 7β-hydroxycholesterol, recognized *in vivo* markers of oxidative stress, was observed in the COVID-19 patients but not in pauci- and asymptomatic patients (88).

Even though knowledge about SARS-CoV-2 entry still grows rapidly, the studies presented and discussed above may help understand the role of cholesterol-rich lipid rafts in this viral replication process (**Figure 1**).

## Cholesterol-Rich Lipid Rafts Provide a Platform to Concentrate SARS-CoV-2 Receptors

Cholesterol-rich lipid rafts serve as a platform for the functional organization of the receptors involved in the cell signaling, synaptic activity, immune response, membrane trafficking, and cytoskeleton remodeling (19, 89–92). The pathogen interplay with cholesterol-rich lipid rafts modulates many cellular processes. During virus entry into the cell, the cholesterol-rich lipid rafts contain receptors and co-receptors that interact with viral surface proteins (31, 93). Moreover, the entry of enveloped and non-enveloped viruses into host cells occurs through fusion or endocytosis mediated by caveolin or clathrin located in the lipid-rich microdomains (94). In this regard, the study of cholesterol-rich lipid rafts is rapidly expanding and has again become an attractive topic for SARS-CoV-2 research. Hence, the role of receptors for SARS-CoV-2 entry localized and distributed on cholesterol-rich lipid rafts during infection will be discussed below (**Figure 1** and **Table 1**).

**Table 1:** Summary of the cellular receptors from cholesterol-rich lipid rafts that are involved in SARS-CoV-2 entry.

Receptor	Cellular function	Proposed entry mechanism	References
ACE-2	ACE-2 is a negative regulator of RAS and a catalyst for converting angiotensin II to angiotensin 1-7. ACE-2 is expressed in various organs such as the heart, lung, kidney, liver, etc.	ACE-2 binds to the S protein-RBD of SARS-CoV-2, facilitating virus entry via caveolin- or clathrin-dependent endocytosis.	(8)
TMPRSS2/4	TMPRSS are vital regulators of mammalian development and homeostasis in different tissues as the liver, lungs, pancreas, intestinal tract, and salivary glands.	TMPRSS2 and TMPRSS4 enhance cellular-virus membrane fusion by inducing protein S cleavage and exposing the fusion peptide, which interacts with the ACE-2 receptor.	(13, 95)
HSPG	HSPG participates in multiple functions such as cellular adhesion and motility; moreover, they serve as receptors for endocytosis and are also involved in the control of numerous events that occur during inflammation	The interaction between SARS-CoV-2 spike protein and HSPG is necessary for the viral entry via endocytosis ACE-2-dependent.	(10, 96, 97)
Syndecan-1/4	Syndecans are expressed in various cellular sites and participate during adhesion between cell and extracellular matrix, cell-cell adhesion, cell migration, and regulation of the inflammatory response.	Syndecan-1/4 interacts with the S1 subunit of SARS-CoV-2 spike protein, an essential viral attachment factor, and mediator of viral entry.	(15, 98)
TLR-4	TLR4 is a key receptor that induces the pro-inflammatory response, can mediate inflammation by both exogenous and endogenous ligands, and is associated with chronic and acute diseases, promoting amplification of the inflammatory response.	TLR4 interacts with the S1 subunit of spike protein and is involved in SARS-CoV-2 entry, even if the cell line lacks the ACE-2 receptor. However, evidence on the mechanism of entry used by the virus is lacking.	(99–102)
CD147	CD147 is a transmembrane glycoprotein member of the immunoglobulin superfamily implicated in various physiological and pathological conditions due to its regulation of cell-cell recognition, cell differentiation, and tissue remodeling.	S protein of SARS-CoV-2 interacts with the CD147 receptor and facilitates virus entry via endocytosis even in the absence of the ACE-2 receptor.	(11, 103)
NRP1	NRP1 is a pleiotropic transmembrane polypeptide that acts as a growth factor or a cofactor in fibroblasts, platelets, hepatocytes, etc.	NRP1 enhances SARS-CoV-2 entry and infectivity only in co-expression with ACE-2 and TMPRSS2.	(53)
L-SIGN	L-SIGN is a type II C-type lectin receptor involved in cell adhesion and pathogen recognition. It is expressed in dendritic cells, epithelial cells, lungs, liver, lymph nodes, and placenta.	L-SIGN binds to high-mannose-type N-glycans present in the spike protein of SARS-CoV-2, favoring the viral entry in the presence of the ACE-2 receptor.	(16, 104)
AXL	AXL is a receptor tyrosine kinase; its activation promotes homodimerization, causing tyrosine autophosphorylation or phosphorylation of downstream targets, activating signaling pathways.	The NTD of the SARS-CoV-2 spike protein binds to AXL, independently of the presence of the ACE-2 receptor. However, low levels of the ACE-2 receptor synergize with the expression of the AXL to potentiate SARS-CoV-2 infection.	(14, 17, 105)
SR-B1	SR-B1 is the cell-surface HDL receptor that mediates a selective uptake system for cholesterol and other lipids in various cells, such as fibroblasts, hepatocytes, macrophages, adrenal, and alveolar cells.	The RBD of the SARS-CoV-2 S protein has an affinity for cholesterol and HDL components, enhancing the entry of the virus into the cell through SR-B1 only when ACE-2 es expressed.	(12, 106)

### Angiotensin-Converting Enzyme-2 (ACE-2) 

The ACE-2 protein, localized in cholesterol-rich lipid rafts, is used as a functional receptor for human coronaviruses (48, 107). ACE-2 is a homolog of ACE-1 receptor that mediates the angiotensin II production to activate the renin-angiotensin system (RAS) and plays a crucial role in cardiovascular diseases (6). Also, the ACE-2 receptor is widely expressed in various organs such as the heart, lung, kidney, and liver (108, 109). The interaction between the host cell and SARS-CoV-2 is closely identical to SARS-CoV, which also uses the ACE-2 as a cellular receptor (**Figure 1**). It facilitates virus entry *via* caveolin- or clathrin-dependent endocytosis. (51, 52). Although the predominant symptoms of SARS-CoV-2 infection are respiratory, multiple organ injuries such as renal and hepatic abnormalities and cardiac lesions are observed among COVID-19 patients (109). This tropism is attributed to the presence of the ACE-2 receptor in various organs (108). However, the fact that the participation of other receptors may enhance the SARS-CoV-2 entry is not ruled out. Recognizing mannosylated N-glycan and O-glycan on the S protein by cellular receptors found in the cholesterol-rich lipid rafts could facilitate the SARS-CoV-2 entry (**Figure 1**) (50). Therefore, the quick adaptation of SARS-CoV-2 to cell receptors could be associated with new pathologies or more severe diseases. In the following, we will describe new evidence on the role of other receptors in SARS-CoV-2 entry.

### Transmembrane Serine Proteases (TMPRSS)

The TMPRSS subfamily includes membrane-anchored serine proteases belonging to the serine protease type II family that possesses an N-terminal transmembrane domain and a C-terminal extracellular chymotrypsin serine protease domain (95). This subfamily comprises seven members: TMPRSS1/hepsin, TMPRSS2, TMPRSS3, TMPRSS4, TMPRSS5/spinesin, mosaic serine protease large-form (MSPL), and enteropeptidase (110). TMPRSS receptors are expressed in various tissues such as the liver, lungs, pancreas, intestinal tract, and salivary glands (95). In addition, TMPRSS are vital regulators of the mammalian development, homeostasis, and host factors involved in the entry of coronaviruses (111).

The TMPRSS2 and TMPRSS4 receptors activate the glycoproteins of influenza virus, SARS-CoV, and MERS-CoV to enhance the viral entry, promoting the syncytia formation and cell tropism since these receptors are expressed in epithelial cells of the respiratory and intestinal tracts (112–115). Interestingly, TMPRSS2 contains a potential palmitoylation residue in the cytoplasmic tail responsible for its localization in cholesterol-rich lipid rafts (116). Moreover, this receptor is associated with ACE-2. Thus, both receptors are membrane-embedded of these microdomains (116). Hence, cellular entry and susceptibility of the coronaviruses can be defined by the expression of both ACE-2 and TMPRSS receptors.

Hoffmann et al. demonstrated that SARS-CoV-2 uses the TMPRSS2 for S protein priming, and that the infection of lung cells with this virus can be blocked by a TMPRSS2 inhibitor (**Figure 1**) (64). TMPRSS2-dependent SARS-CoV-2 entry may be due to precleavage of the furin-dependent Subunit1/Subunit2, which is essential for the releasing viral RNA into the cell cytoplasm (64). Zang et al. described that both TMPRSS2 and TMPRSS4 enhance membrane fusion by inducing S protein cleavage, exposing the fusion peptide in gastrointestinal tract cells after binding to the ACE-2 receptor (13). Also, TMPRSS4, one of the most significantly correlated genes with the ACE-2 receptor expression (117), enhanced the SARS-CoV-2 entry into human small intestinal enterocytes, while some COVID-19 patients shed high levels of viral RNA in feces (13). However, the SARS-CoV-2 particles released in the feces are rapidly inactivated by the low pH of gastric fluids, consistent with the previous reports on SARS-CoV and MERS-CoV infections (13). These findings suggest that the intestine is a potential site of SARS-CoV-2 replication, where TMPRSS4 plays an important role, contributing to local and systemic disease and gastrointestinal symptoms progression (13).

The salivary glands from SARS-CoV-2-infected patients have the ACE-2 and TMPRSS receptors overexpression (118). Also, the ultrastructural analysis of the ductal lining cell cytoplasm, acinar cells, and ductal lumen was performed by electron microscopy, a tool used to identify viral particles (119), revealed coronavirus-like particles (118). These findings demonstrated that the salivary glands are a reservoir for SARS-CoV-2, supporting the use of the saliva as a diagnostic method for COVID-19 and the role of this biological fluid in spreading the disease (118). TMPRSS3, TMPRSS4, TMPRSS5, and TMPRSS7 correlate with the ACE-2 expression in salivary glands, and these receptors are overexpressed when the basal cells in the oral cavity are differentiated to suprabasal cells (120). However, this cellular differentiation prepares the cells to shed from the oral cavity (121). Furthermore, the stratified squamous cells of the oral cavity are not linked to specific symptoms and shed continuously in the saliva, which could result in asymptomatic COVID-19 patients (120, 121).

The proteolytic activation of S protein was insufficient to fuse the viral membrane to the cell that does not express TMPRSS receptor, confirming the importance of TMPRSS in SARS-CoV-2 entry (122). In contrast, the SARS-CoV-2 S protein was activated, exposing its fusion peptide and facilitating early penetration of the virus into the cytosol, pH-independent, in the cells expressing TMPRSS2 (122). Thus, TMPRSS2 expression dictates the entry route of SARS-CoV-2 to infect the host cells and could have implications in the adaptation and expanded tropism of the virus (122).

The SARS-CoV-2 S1/S2 cleavage site is mutated when the virus is serially passaged in TMPRSS2-deficient cells (123). This event led to a loss of sensitivity to the TMPRSS2 receptor (123). Also, this mutation prevents direct fusion mediated by TMPRSS2, showing a narrow range of the cell tropism (123). Therefore, more attention should be paid to Vero cells in the isolating and propagating SARS-CoV-2, developing vaccines, and *in vitro* evaluation of the antiviral activity of drugs against this virus due to the possibility that some viral genomes accumulate mutations in the S gene by the absence of the TMPRSS2 receptor (123). In summary, TMPRSS receptors located in cholesterol-rich lipid rafts are critical host factors involved in the mechanisms of SARS-CoV-2 entry. They can be an essential study target to understand the pathogenicity of COVID-19.

### Heparan Sulfate

Heparan sulfate proteoglycans (HSPG) are linear sulfated polysaccharides found on the cholesterol-rich lipid rafts (97). HSPG participates in the cellular adhesion and motility, endocytosis, and the control of numerous events during inflammation (96, 97). Due to the negative charge attributed by the sulfated chains, HSPG can interact electrostatically with the basic residues of viral glycoproteins and capsid, favoring the interaction between the viral particle and their specific entry receptor (124). HSPG facilitates initial viral particle-host cell interactions with influenza virus, dengue virus, herpes virus, and some human coronaviruses (124).

The S protein from NL63 and SARS-CoV binds to HSPG, an important co-receptor that facilitates virus internalization. The sialic acid is used as an attachment factor for MERS-CoV entry (125–127). The SARS-CoV-2 S protein binding to the ACE-2 receptor is an HSPG-dependent pathway and necessary for efficient viral replication (**Figure 1**) (10). Interestingly, the RBD of the SARS-CoV-2 S protein can bind HSPG in a length- and sequence-dependent manner (128). This interaction can be drastically reduced by treating heparin lyases that degrades cell-surface HSPG (10). Additionally, a variety of HSPGs may further modulate the tissue tropism and susceptibility to COVID-19 in the population (10).

### Syndecans 

Syndecans are membrane surface proteins that belong to the proteoglycan family, located in cholesterol-rich lipid rafts (129). However, syndecans can be found at various cellular sites, containing other glycosaminoglycan structures and participating in adhesion between cell-extracellular matrix, cell-cell, cytoskeleton-syndecan proteins, and cell migration (98, 129). Syndecan-1 can mediate the inflammatory response by binding to inflammation-related factors, negatively regulating leukocyte migration and adhesion, and modulating the cytokine gradient activity (130).

Syndecan-4 negatively modulates the activation of the antiviral immune response, inhibiting especially the type 1 interferon response (IFN-1) induced by retinoic acid-induced gene 1 (RIG-1) (131). Syndecans are involved in the viral attachment of other viruses (132–135).

**Figure 2:**
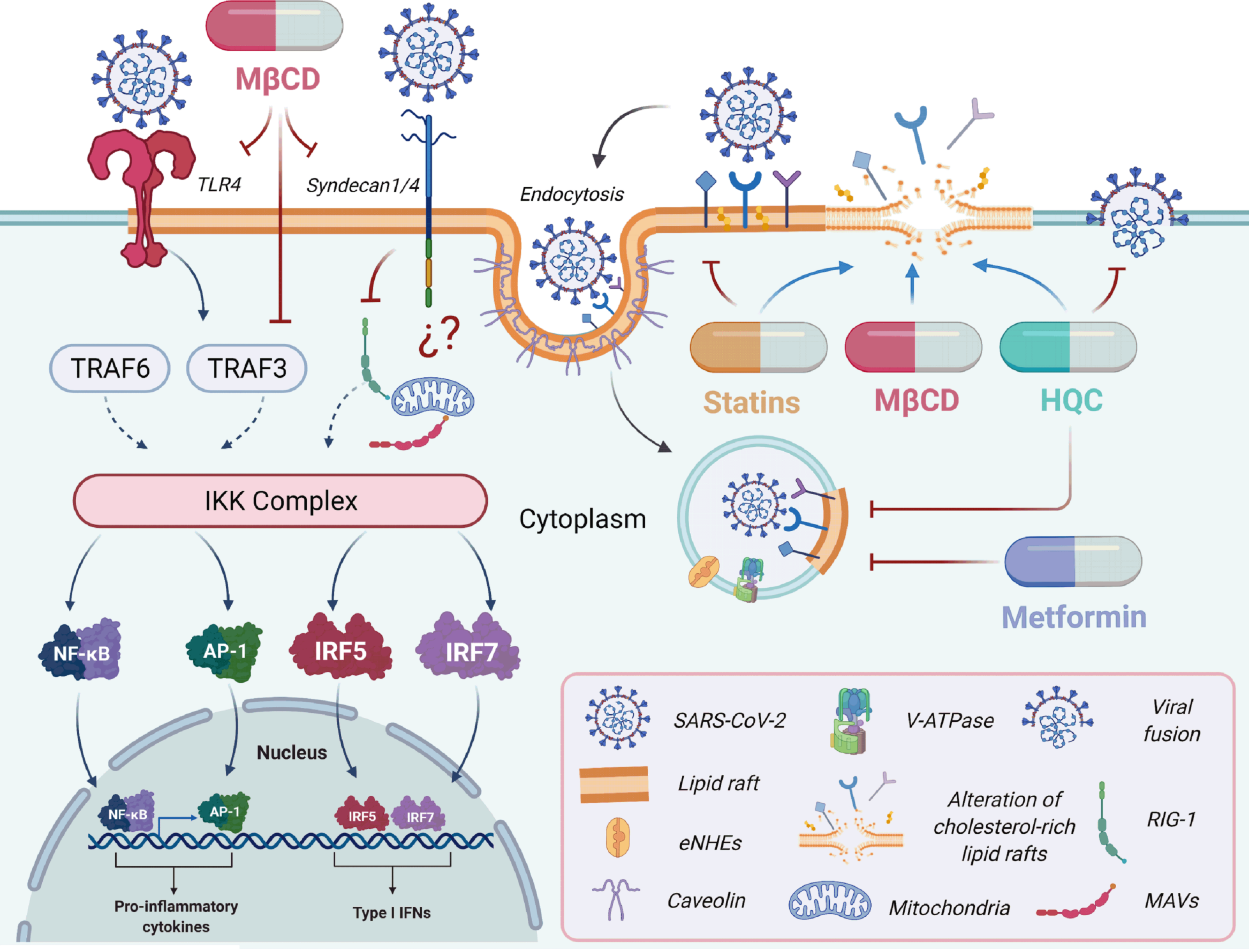
The potential therapeutics of drugs targeting cholesterol-rich lipid rafts in SARS-CoV-2 infection. Therapeutic strategies to inhibit viral replication, including the use of lipid-lowering drugs as antivirals candidates, are based on the study of lipids and their importance in the viral cycle (139). The lipid raft microdomains are primarily associated with the viral entry and play an essential role during other viral cycle stages, such as cellular signal transduction. SARS-CoV-2 entry depends on binding to ACE2; other receptors such as TLR4 or Syndecan 1/4 are involved in pro-inflammatory cytokines (140) and inflammation response (141). Interestingly, cholesterol depletion of lipid rafts using cholesterol-lowering treatments such as methyl-β-cyclodextrin (MβCD) (42, 59), statins (41), and hydroxychloroquine (HQC) (142) affect the interaction between the SARS-CoV-2 spike protein and the ACE-2 receptor. Metformin (143) and HQC (144) can increase the pH values of endosomes acting on the Vacuolar ATPase (V-ATPase) and endosomal Na+/H+ exchangers (eNHEs). This mechanism inhibits the viral infection by increasing the cellular pH and interfering with the endocytic cycle (143, 145, 146). The graphical was elaborated using BioRender.com.

Due to the role of syndecans in viral infections, Hudák et al. explored the possible interactions between SARS-CoV-2 S protein and the isoforms of syndecans, identifying that syndecan-3 and -4 facilitated the uptake of SARS-CoV-2 (**Figure 1**), and syndecan-4 specifically interacts with the S1 subunit of the S protein to mediate SARS-CoV-2 internalization (15). Moreover, syndecan-1 mediates cell attachment of S protein in lung epithelial cells (136). The hypoxia could modulate the expression of ACE-2 and syndecan-1 receptors, suggesting that low oxygen levels in COVID-19 patients are a defense mechanism to reduce the expression of entry receptors and attachment factors located in cholesterol-rich lipid rafts (136). Notably, the distribution of syndecan-4 is ubiquitous, and its expression is abundant in the lungs, one of the target organs of SARS-CoV-2 (137). Syndecan-4 would enhance the virus entry into lung epithelial cells and the modulation of the immune response, contributing to the pathogenesis and the severe lung damage in SARS-CoV-2-infected patients (138). The crucial role of syndecan receptors in SARS-CoV-2 entry is evident; however, it remains to be elucidated whether syndecan-1 and -4 in SARS-CoV-2 entry can modulate negatively or positively the signaling of the antiviral immune response (**Figure 2**).

### Human Toll-Like Receptors (TLRs)

TLRs are a family of integral membrane glycoproteins involved in the innate immune response (147). TLR possesses a leucine-rich extracellular region that interacts with a ligand such as pathogen-associated molecular patterns (PAMPs) from various pathogens, including bacteria, fungi, parasites, and viruses, promoting receptor dimerization and subsequent recruitment signaling molecules (147). The TLR family comprises 12 members, which are expressed in distinct cellular compartments. TLR1, TLR2, TLR4, TLR5, and TLR6 are expressed on the cell surface, whereas TLR3, TLR7, TLR8, and TLR9 are expressed in vesicles and the endoplasmic reticulum intracellularly (148). The TLRs are localized in the cholesterol-rich lipid rafts on the cell surface (101). In this place, the cyclodextrins (MβCD) can sequester cholesterol, affecting the activation of inflammatory response and cytokine secretion and chemokines (101). In this regard, the proteins related to vesicular trafficking, such as the Soluble *N*-Ethylmaleimide-Sensitive Factor Attachment Protein Receptor (SNARE), syntaxin 4, and SNAP-23, are clustered in cholesterol-rich lipid rafts where they participate in cytokine release (149, 150). When the cholesterol-rich lipid rafts localized in the macrophage plasma membrane are disrupted, the phagocytic cup formation is altered, and TNF secretion is reduced (149, 150). During SARS-CoV-2 infection, cholesterol depletion of lipid rafts with MβCD could affect the TLR clusters and the effective activation of kinase complexes (IKK) (**Figure 2**) (59). It could also have an impact on the transcription factors (Nuclear factor-kappa β (NF-kB) and Interferon regulatory factor (IRF), involved in the activation of genes related to pro-inflammatory response and secretion of cytokines and chemokines (**Figure 2**) (101). TLR4 is a key receptor that induces the pro-inflammatory response by exogenous and endogenous ligands and is associated with chronic and acute diseases, promoting amplification of the inflammatory response (100).

The viral glycoproteins or capsid proteins can interact with TLR4 to activate its signaling pathway and an uncontrolled inflammatory response, leading to virus disease severity (151). Interestingly, the TLR4-mediated signaling pathway could benefit multiple steps of the virus life cycle, such as enhancing viral particle release from the cell or preventing premature cell apoptosis, since cell survival factors can also be activated *via* TLR4 (151).

Due to the acute inflammation in SARS-CoV-2 pathogenesis, Choudhory et al. performed an *in silico* analysis of the interaction between the S protein and cell surface receptors of the innate immune response, especially TLRs. Choudhory et al. demonstrated by molecular docking a significant binding between the S protein and TLR1, TLR4, or TLR6, of which the SARS-CoV-2 S protein-TLR4 interaction possessed the strongest binding energy (**Figure 2**) (140). Even though experimental evidence confirming the interaction between the S protein and TLRs is lacking, the main cytokines involved in patients with severe COVID-19 are products of TLR4 viral signaling: IL-6 and TNF-α (**Figure 2**) (140). In addition, the S1 subunit of S protein promotes TLR4 activation inducing the expression and secretion of TNF-α mRNA, which was significantly suppressed with a TLR4 antagonist (**Figure 2**). Curiously, this effect was not observed when a TLR2 antagonist was used. Thus, SARS-CoV-2 S protein-TLR4 interaction is involved in the inflammatory response triggered by COVID-19 (102). Also, this interaction would promote endocytosis of the virus and activation of the TLR4 signaling cascade to trigger the pro-inflammatory response (**Figure 2**) (101). SARS-CoV-2 can infect the cerebral cortical neurons, cells that do not express ACE-2 receptor but contain TLR4 receptor, and activate the pro-inflammatory response, suggesting that the TLR4 is involved in SARS-CoV-2 entry and could be related to the neurological manifestations of COVID-19 (99). Further studies are needed to clarify the involvement of TLR4 as a co- or receptor for SARS-CoV-2. Moreover, TLRs are involved in the activation of the immune response causing a cytokine storm in COVID-19 patients, where TLR-3, -7, -8 through viral RNA recognition triggers the activation of JAK/STAT, NF-kB, AP-1 signaling pathways resulting in the amplification of pro-inflammatory cytokines (152, 153). Thus, TLR7/8 antagonist drugs (Hydroxychloroquine, HCQ; a TLR blocker) could limit SARS-CoV-2 infection (152, 153). In this sense, HCQ can inhibit endosomal TLR3, -7, -8, and -9 signaling, controlling inflammation in COVID-19 patients and mitigating the detrimental effects of viral infection (152, 153).

### Dendritic Cell-Specific Intercellular Adhesion Molecule-3-Grabbing Nonintegrin Related (L-SIGN or DC-SIGNR)

Also known as CD209L, L-SIGN is a type II C-type lectin receptor found in cholesterol-rich lipid rafts and expressed in dendritic cells, epithelial cells, lungs, liver, lymph nodes, and placenta (104). L-SIGN can bind to high mannose oligosaccharides through its carbohydrate recognition domain, and it is involved in the attachment of viral glycoproteins (104). L-SIGN participates in SARS-CoV infection and pathogenesis, where the presence of ACE-2 is required for efficient virus entry into the cell (154). Thus, the role of L-SIGN as a co-receptor enhances viral entry (154). Interestingly, a genetic risk association study revealed that individuals homozygous for the CD209L receptor tandem repeats were less susceptible to SARS-CoV infection (155). Hence, the ligand-binding capacity dependent on homo- or heterozygosity of L-SIGN plays a protective role in affecting the susceptibility to SARS-CoV infection (155).

L-SIGN is an endothelial receptor for SARS-CoV-2 that could contribute to the COVID-19 associated coagulopathy (16). Notably, high-mannose-type N-glycans present in the SARS-CoV-2 S protein play a decisive role in the binding to L-SIGN (**Figure 1**), suggesting that blockade of L-SIGN would serve as a novel antiviral therapy option (16). The SARS-CoV-2 mutation at the D614G glycosylation site is one of the more infectious dominant variants in the early phases of the pandemic (156). In this regard, this mutation raises the glycosylation of S protein, enhancing virus entry into the cell, contributing to the severity of SARS-CoV-2 infection (156, 157).

### AXL Receptor Tyrosine Kinase (AXL)

AXL is localized in cholesterol-rich lipid rafts, and its activation promotes homodimerization, causing tyrosine auto- or phosphorylation of downstream targets (105). AXL is expressed ubiquitously in several cell types, and its function depends on the specific cell/tissue type (105). This receptor participates in (1) virus binding and internalization; and (2) viral replication by antagonizing the IFN-1 pathway (158). The AXL receptor, expressed on lung epithelial cells specifically, can bind to the SARS-CoV-2 spike protein through their N-terminal domain to promote viral entry, independently of the presence of the ACE-2 receptor (**Figure 1**) (17). Therefore, the interaction between the SARS-CoV-2 S protein and the AXL receptor may support an essential role of AXL during infection of human pulmonary and bronchial tissues (17). In addition, low levels of the ACE-2 receptor synergize with the expression of the AXL receptor to potentiate SARS-CoV-2 infection (14). Together, these studies confirm that the AXL receptor is essential in SARS-CoV-2 entry into lung cells, and this mechanism could be effectively disrupted in human lung cells by the AXL inhibitors.

### High-Density Lipoprotein (HDL) Scavenger Receptor B Type 1 (SR-B1)

SR-B1 is the cholesterol-rich lipid rafts HDL receptor that mediates a selective uptake system for cholesterol and other lipids in various cells, such as fibroblasts, hepatocytes, macrophages, adrenal, and alveolar cells (106). The RBD of the SARS-CoV-2 S protein has a particular affinity for cholesterol and HDL components, enhancing the virus entry into the cell through SR-B1, suggesting that this interaction is dependent on the presence of membrane cholesterol (**Figure 1**) (12). In addition, ACE-2 and SRB-1 are co-expressed in multiple susceptible tissues (159). Therefore, this evidence further supports the important role of cholesterol-rich lipid rafts and receptors that regulate lipid entry into the cell to enhance the SARS-CoV-2 entry.

### Other Immune Receptors

**CD147** is a member of the immunoglobulin superfamily, located in cholesterol-rich lipid rafts (103). CD147 facilitates SARS-CoV infection (160). It can bind to SARS-CoV-2 S protein to promote the endocytosis-dependent viral internalization even in the absence of the ACE-2 receptor, revealing a novel virus entry route (11).

Ahmetaj et al reported that the cardiorenal tissues and endothelial cells express the CD147 and ACE-2 genes required for SARS-CoV-2 entry (**Figure 1**) (161). Interestingly, ACE-2 decreases with age in some tissues, and CD147 increases with age in endothelial cells, suggesting that CD147 expression in the vasculature may explain the heightened risk for COVID-19 severe with age (161). Moreover, CD147 is expressed in the kidney of COVID-19 patients, where its distribution is expanded from the basolateral to the circumferential pattern, including interfacial and apical sides (162). Thus, CD147 apical presentation likely contributes to SARS-CoV-2 internalization from that lumen side into the cytoplasm of tubular epithelial cells (162).

**Neuropilin-1** (**NRP1)** is a pleiotropic transmembrane polypeptide that acts as a growth factor or cofactor in fibroblasts, platelets, and hepatocytes (163). NRP1 is involved in the SARS-CoV-2 infection, and in contrast with ACE-2 and TMPRSS2 receptors, NRP1 does not enhance SARS-CoV-2 entry in cell lines that only express this receptor (53). However, when NRP1 is co-expressed with ACE-2, and TMPRSS2 the SARS-CoV-2 infection significantly increases, defining its role as an essential co-receptor for viral entry (**Figure 1**) (53). Since there is limited evidence, further studies are needed to clarify the interaction between the SARS-CoV-2 S protein and NRP1 receptor and how it facilitates viral entry (50, 53).

## Targeting Cholesterol-Rich Lipid Rafts as Potential Therapeutics in SARS-CoV-2 Infection 

Lipidomic evidence suggests a remodeling of lipid metabolism in coronavirus-infected human cells (164). This alteration is associated with aberrant lipid metabolism in obese patients who develop a decreased immune response, which increases the severity of COVID-19 (165). Cholesterol localized in the lipid rafts is an essential entry factor for coronaviruses, both *in vitro* and *in vivo* (42, 48, 166), and a determinant of the SARS-CoV-2 pathogenesis and replication (167).

Therapeutic strategies to inhibit viral replication, including the use of lipid-lowering drugs as antivirals candidates, are based on the study of lipids and their importance in the viral cycle (139). Although, as previously discussed, this review is focusing on cholesterol-rich lipid rafts and the SARS-CoV-2 entry (9–17), the essential role of cholesterol during SARS-CoV-2 replication and egress cannot be ruled out (71, 85, 168–171). It is essential to mention here that although a notable amount of work has been carried out on the relationship between SARS-CoV-2 entry and lipid rafts, few studies have been published focusing on the role of the lipid rafts in SARS-CoV-2 replication and egress.

Cholesterol is an essential component of host cell membranes involved in tuning membrane fluidity, thickness, and permeability to regulate membrane function (172). The viral replication complexes are RNA virus-induced membrane structures where viral genome replication and morphogenesis occur (119). The formation of the replication complex requires cholesterol, a product of fatty acid metabolism (26, 173). Williams et al. demonstrated that the inhibition of fatty acid metabolism by orlistat [Food and Drug Administration (FDA)-approved drug that inhibits gastric lipases and fatty acid synthase (FASN)], TOFA [a competitive inhibitor of acetyl-CoA carboxylase (ACC)], A922500 (a potent inhibitor of diacylglycerol acyltransferase 1 (DGAT1) or VPS34-IN1 [an inhibitor of vacuolar protein sorting 34 (VPS34-IN1)], interferes with the formation of dsRNA-positive SARS-CoV-2 replication complexes (171). Transmembrane protein 41B (TMEM41B) likely contributes to SARS-CoV-2 replication complexes formation through cholesterol trafficking to facilitate host membrane expansion and curvature (173).

Lipid droplets store neutral lipids and cholesterol, and they are a platform for SARS-CoV-2 assembly and replication (174). Additionality, modulation of lipid droplets formation by inhibition of DGAT1 using A922500 can block SARS-CoV-2 replication and reduce the production of mediators pro-inflammatory response (171, 174).

On the other hand, Daniloski et al. identified a group of host genes (RAB7A, NPC1, ATP6AP1, ATP6V1A, CCDC22, and PIK3C3) implicated in the upregulation of the cholesterol synthesis pathway during SARS-CoV-2 infection (71). A parallel genome-scale CRISPR-Cas9 knockout screen revealed genes involved in sensing and biosynthesis of cholesterol, such as Sterol Regulatory Element Binding Transcription Factor 2 (SREBF2) and SREBP cleavage activating protein (SCAP) which are required for SARS-CoV-2 infection (170). This finding agrees with Hoffmann et al., who performed a focused high-coverage CRISPR-Cas9 library targeting 332 host proteins identified as high-confidence SARS-CoV-2 protein interactors (175). Interestingly, Wang et al. also identified clusters linked to cholesterol metabolism (low-density lipoprotein receptor (LDLR), NPC1, SCAP, and SREBF2) as a critical host pathway through a genome-wide CRISPR screen in SARS-CoV-2-infected cells (176). The SREBP family of transcription factor control cholesterol and lipid metabolism (177). The treatment with SREBP pathway modulators such as PF-429242, 25-HC, and Fatostatin reduces the SARS-CoV-2 replication and entry, suggesting that cellular cholesterol is required (176). Also, amlodipine, a calcium-channel antagonist, increases cholesterol levels and blocks SARS-CoV-2 infection (71). This finding is consistent with the Zhang et al. study, where amlodipine and other calcium channel inhibitors blocked the post-entry replication events of SARS-CoV-2 *in vitro* (178). Zhang et al. associated the amlodipine therapy with a decreased case fatality rate in COVID-19 patients (178). Also, a cholesterol accumulation by treating 25-HC and NPC1 inhibitors itraconazole (ICZ) and U18666A restricts SARS-CoV-2 replication (85).

Some FDA-approved cholesterol-lowering drugs have antiviral properties and are safe for use in humans, which reduces the time and requirements for their study in clinical trials (179). In this regard, statins and metformin are promising candidates for the treatment of infections caused by enveloped viruses, such as Dengue virus (DENV), Zika virus (ZIKV), hepatitis C virus (HCV), Japanese encephalitis virus (JEV), influenza A virus (IAV), and recently for the treatment of SARS-CoV-2 (180–184). These drugs interfere in different metabolic pathways for lipid synthesis by inhibiting critical cholesterol and fatty acid synthesis (185).

Statins directly inhibit the HMGCR enzyme, responsible for *de novo* cholesterol synthesis inducing alteration of cholesterol-rich lipid rafts (**Figure 2**) (186, 187) and, by a consequence, inhibits infection caused by coronaviruses (41). Interestingly, the use of statins is associated with a lower risk of mortality among people with COVID-19 (188, 189); however, its use to treat these diseases remains controversial (190–192). Although the antiviral mechanism of statins is unknown, it could affect viral replication and morphogenesis, as occurs with other viruses (193–196). Furthermore, the immunomodulatory properties of statins are another advantage for treating viral diseases, such as those caused by influenza and Ebola viruses (182, 197, 198).

Metformin is another drug with lipid-lowering effects that have gained interest in recent decades due to its pleiotropic effects and antiviral properties (199). Metformin inhibits cholesterol and fatty acid synthesis by activating the AMP-activated protein kinase (AMPK), involved in multiple energetic pathways in the cell (200). Similar to statins, its lipid-lowering effect, coupled with the immunomodulatory effects of Metformin, could be responsible for the benefits reported in COVID-19 patients with type 2 diabetes and insulin resistance (201–203). Thus, the use of Metformin could benefit the survival of older adults infected by SARS-CoV-2 compared to those who do not take this drug (204–206). Metformin could increase the endosomal and lysosomal pH values. Acting directly on two crucial membrane compartments found in cholesterol-rich lipid rafts to maintain and regulate the endosomal acidic pH (**Figure 2**): (1) using the Vacuolar ATPase (V-ATPase) as a proton-pumping or acidifier compartment; (2) following the endosomal Na+/H+ exchangers (eNHEs), as proton leaking or alkalizing compartment on the endosomal membrane (143). These mechanisms lead to the inhibition of viral infection through increasing the cellular pH and subsequently interfering with the endocytic cycle (143, 145, 146). In addition, metformin and the fatty acid synthase (FASN) inhibitor orlistat can inhibit coronavirus replication and reduce systemic inflammation to restore immune homeostasis (165).

SARS-CoV-2 entry depends on binding to ACE2 localizes to both monosialotetrahexosylganglioside1 (GM1) lipid rafts and PIP2 domains embedded in cholesterol-rich lipid rafts (141). Drugs such as HC directly perturb ordered GM1 (142), inhibiting viral entry by alteration of the cholesterol-rich lipid rafts where the SARS-CoV-2 receptors are located (**Figure 2**) (142, 144). Another effect is the capability to negatively alter endocytosis, maturation of endosomes, and transport virions (**Figure 2**) (144). Moreover, cholesterol depletion of membranes with MβCD reduces the SARS-CoV-2 infection (59). All these reports confirm the role of cholesterol-rich lipid rafts as therapeutic targets for COVID-19.

At present, there is no scientific evidence that treatment with these drugs can worsen covid-19 disease; on the contrary, it may improve the outcome of SARS-COV2-infected patients (**Table 2**). Therefore, the risk of their use is limited to the side effects already known for each drug (**Table 2**) (188, 189, 204–208). Regarding statins, some of the side effects increased the incidence of diabetes and cataracts and frequent muscular side effects (209, 210). In the case of metformin, the main side effect is lactic acidosis (211). It should be noted that their use as antivirals suggests an acute and short-term treatment, reducing the side effects associated with long periods of treatment (209, 211–213). Preclinical studies are necessary to evaluate its safety during viral infections, as currently metformin is evaluated during ZIKV infections (184, 214, 215).

**Table 2:** Anti-SARS-CoV-2 activity of FDA-approved cholesterol-lowering drugs.

Lipid-lowering Drug	Study type	Effect	References
**ATV, RSV, SIM, PRV, FLV and PTV**	Retrospective study: a. 13,981 patients diagnosed with SARS-Cov-2 in Hubei Province, China b. 2921 patients diagnosed with SARS-Cov-2, who are hospitalized in 150 Spanish hospitals.	Reduced risk of mortality among people with COVID-19	(188, 189)
**MET**	Retrospective studies: a. 283 diabetic patients hospitalized with confirmed SARS-Cov-2 in the Tongji Hospital of Wuhan, China. b. 1139 patients positive SARS-Cov-2 in 8 states in USA. c. 775 nursing Home Residents Infected with SARS-CoV2 from the Community Living Centers (CLC), USA.	a. Antidiabetic treatment with metformin was associated with lower hospitalization and mortality. b. Relative survival benefit in nursing home residents on metformin.	(204–206)
**CQ/HCQ**	Clinical study: a. Treating group of 100 COVID-19 patients treated with CQ. b. 36 patients diagnosed with SARS-Cov-2. Treatment group (20 patients) received HCQ 200 mg for ten days, three times a day (600 mg daily). Control group (16 patients). Six patients received AZI to prevent bacterial infections.	a. Improvements of pneumonia and lung imaging and reduction of the duration of illness without any adverse effects. b. On day six, treatment group showed a significant reduction in the viral load. The six patients who received a combination (HCQ and AZI) were testing negative, indicates the high effectiveness of the combination.	(207, 208)

SARS-CoV-2, severe acute respiratory syndrome coronavirus; STAs, Statins; ATV, Atorvastatin; RSV, Rosuvastatin; SIM, Simvastatin; PRV, Pravastatin; FLV, Fluvastatin; PTV, Pitavastatin; MET, Metformin; HCQ, Hydroxychloroquine; AZI, Azithromycin; CQ, Chloroquine.

On the other hand, empirical evidence for HCQ effectiveness in COVID-19 is limited. Currently, a few studies reported the antiviral activity of HCQ against SARS-CoV-2 (144, 216). Following the promising results, the usage of HCQ for certain COVID-19 patients improve. However, HCQ is well known to have severe complications and side effects in some cases. Reports raise concerns that SARS-CoV-2 causes liver and renal impairment, and using HCQ for COVID-19 treatment might increase the risk of toxicity (217). Despite these, clinical trials currently investigate the effectiveness of HCQ in treating COVID-19 (218) because it confers antiviral and anti-inflammatory effects with fewer side effects. However, proper randomized controlled trials of HCQ and individual immune profiles of COVID-19 patients are needed and should be thoroughly evaluated and considered (144).

## Concluding Remarks

It is well known that coronaviruses interact with a large and diverse repertoire of receptors located on lipid rafts, which are regions on the membrane that provide a platform that concentrates receptors that serve as an entry portal into the cell. This review focuses on the role of cholesterol-rich lipid rafts as a platform for SARS-CoV-2 entry. Cholesterol is vital in the SARS-CoV-2 entry and pathogenesis. Several reports demonstrated that deprivation of cellular cholesterol significantly affects SARS-CoV-2 attachment and internalization due to a redistribution of receptors and co-receptors found in the cholesterol-rich lipids rafts, which would attenuate COVID-19 symptoms. Therefore, deciphering the SARS-CoV-2 receptors in cholesterol-rich lipid rafts is vital for developing antiviral strategies that inhibit viral replication.

## Publisher’s Note

All claims expressed in this article are solely those of the authors and do not necessarily represent those of their affiliated organizations or those of the publisher, the editors, and the reviewers. Any product that may be evaluated in this article, or claim that may be made by its manufacturer, is not guaranteed or endorsed by the publisher.

## Author Contributions

All authors included in the manuscript have made substantial contributions to the work. Under the supervision of JR-R, SP-R, RÁ, and VB-D. JR-R, and SP-R: conceptualization. JR-R, SP-R, CC, CF, and LJ-G: investigation and writing-original draft. JR-R, SP-R, VB-D, RÁ, AM-P, and GM-M: formal analysis. JR-R, SP-R, JO-R, GM-M, JQ-G, AM-P, VB-D, and RÁ: writing-review and editing. All authors contributed to the article and approved the submitted version.

## Funding

This research was supported by CONACYT (Mexico), grants: Pronaii 302979 and A1-S-9005 from RA. CF, CC, JO-R, LJ-G, and SP-R had a scholarship granted by CONACYT during the writing of this review.

## Conflict of Interest

The authors declare that the research was conducted in the absence of any commercial or financial relationships that could be construed as a potential conflict of interest.

## Publisher’s Note

All claims expressed in this article are solely those of the authors and do not necessarily represent those of their affiliated organizations, or those of the publisher, the editors and the reviewers. Any product that may be evaluated in this article, or claim that may be made by its manufacturer, is not guaranteed or endorsed by the publisher.
